# Regional Perivascular Space Burden in Pediatric Idiopathic Intracranial Hypertension: A Case–Control MRI Study

**DOI:** 10.3390/diagnostics16203317

**Published:** 2026-10-13

**Authors:** Seda Kaynak Sahap, Eda Almus, Ömer Bektaş, Serap Teber, Suat Fitoz

**Affiliations:** 1 Department of Pediatric Radiology, Faculty of Medicine, Ankara University, 06590 Ankara, Türkiye; edaalmus@gmail.com (E.A.); sfitoz@yahoo.com (S.F.); 2 Department of Pediatric Neurology, Faculty of Medicine, Ankara University, 06590 Ankara, Türkiye; bektasomer@gmail.com (Ö.B.); seraptteber@gmail.com (S.T.)

**Keywords:** idiopathic intracranial hypertension, glymphatic system, perivascular spaces, MRI, pediatric

## Abstract

**Background/Objectives:** Altered perivascular fluid dynamics and glymphatic function have been implicated in the pathophysiology of idiopathic intracranial hypertension (IIH), but data in pediatric patients remain limited. Our aim was to evaluate MRI-visible perivascular space (PVS) burden in pediatric IIH and investigate its relationship with conventional MRI findings, transverse sinus stenosis (TSS), and cerebrospinal fluid (CSF) opening pressure. **Methods:** In this retrospective case–control MRI study, 46 pediatric patients with clinically confirmed IIH and 46 controls frequency-matched by age and sex were included. PVS burden was independently assessed by two pediatric radiologists on axial T2-weighted images using the Potter grading system. Conventional MRI findings associated with IIH and TSS were evaluated. Associations between PVS burden, TSS, and CSF opening pressure were analyzed. Interobserver agreement was assessed using Cohen’s kappa statistics. **Results:** Basal ganglia PVS scores were significantly higher in pediatric IIH patients than in controls (*p* = 0.005), while no significant differences were observed in centrum semiovale or brainstem PVS scores. Centrum semiovale PVS scores differed according to TSS status; TSS-negative patients had lower scores than both TSS-positive patients and controls (Holm-adjusted *p* = 0.007 for both comparisons), whereas TSS-positive patients did not differ significantly from controls. No significant correlation was observed between CSF opening pressure and PVS scores in any evaluated region. Interobserver agreement for PVS grading was good for the centrum semiovale and basal ganglia and moderate for the brainstem. **Conclusions:** Pediatric IIH was associated with increased basal ganglia PVS burden, suggesting a regionally heterogeneous pattern. Pediatric IIH was associated with increased basal ganglia PVS burden, suggesting a regionally heterogeneous pattern. Centrum semiovale PVS burden differed according to TSS status, with lower scores in TSS-negative patients than in both TSS-positive patients and controls, whereas TSS-positive patients did not differ significantly from controls.

## 1. Introduction

Idiopathic intracranial hypertension (IIH), also known as pseudotumor cerebri, is a neurological disorder characterized by elevated intracranial pressure (ICP) of unknown etiology in the absence of intracranial lesion, hydrocephalus, or central nervous system infection [1,2]. Although pediatric IIH shares clinical features with the adult form—such as headache, visual disturbances, and papilledema—its clinical and biological characteristics may differ from those of adult IIH [3,4].

Recent research has increasingly focused on the glymphatic system, a brain fluid transport pathway thought to facilitate the exchange of cerebrospinal fluid (CSF) and interstitial fluid through perivascular spaces (PVS) [5,6]. Dysfunction of this system has been proposed as a potential contributor to the pathogenesis of IIH [2,6,7,8,9,10]. Previous studies in adults have demonstrated a significantly increased burden of magnetic resonance imaging (MRI)-visible PVS in patients with IIH compared to healthy controls, supporting the hypothesis of glymphatic impairment [11]. However, pediatric studies are limited and findings remain inconsistent. A recent pediatric case–control study reported no significant differences in PVS burden or regional diffusivity between IIH patients and controls, suggesting that the relationship between elevated ICP and glymphatic alterations in children may differ from that observed in adults [12].

In contrast to PVS burden, the diagnostic value of established radiological markers-including empty sella, optic nerve sheath distension, posterior globe flattening, optic disc protrusion, and transverse sinus stenosis (TSS)-has been well demonstrated in pediatric IIH [7,13,14]. Nevertheless, the relationship between PVS burden and venous outflow abnormalities, particularly TSS, has not been sufficiently investigated in children.

Therefore, we aimed to compare PVS burden between pediatric patients with IIH and age- and sex-matched controls and to investigate the relationship between PVS burden, conventional MRI findings, TSS, and CSF opening pressure, providing further insight into the potential involvement of glymphatic pathways in pediatric IIH.

## 2. Materials and Methods

### 2.1. Study Design and Population

This retrospective observational study included pediatric patients diagnosed with IIH between March 2015 and March 2026. Brain MRI and, when available, magnetic resonance venography (MRV) examinations of 46 patients were independently reviewed by two pediatric radiologists. All MRI examinations represented the initial diagnostic imaging obtained before the initiation of IIH-specific medical or surgical treatment. This retrospective study was approved by the institutional ethics committee, and the requirement for informed consent was waived because of the retrospective study design.

**Figure 1:**
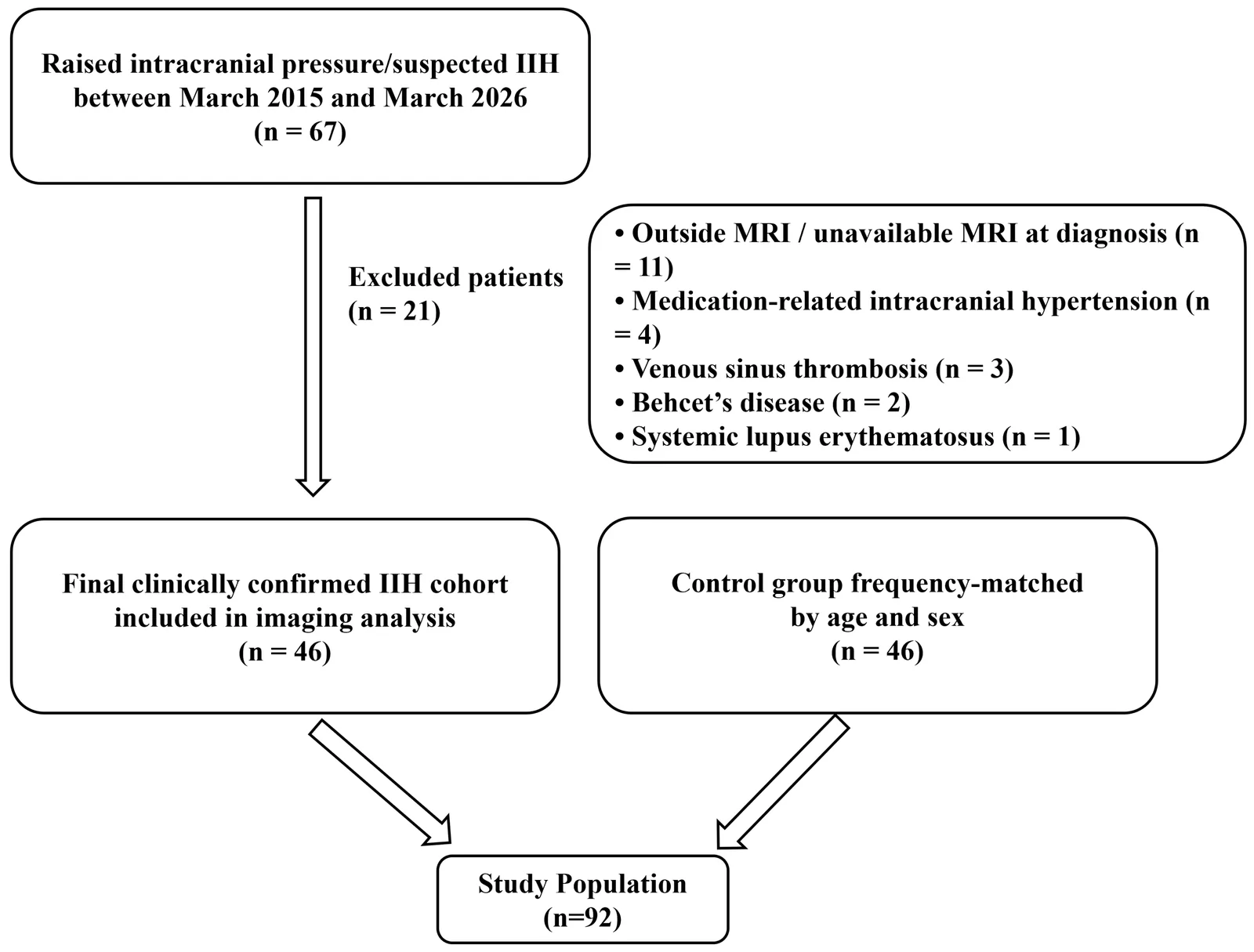
Flowchart of participant selection for the pediatric IIH and control groups.

Inclusion criteria were as follows: age under 18 years, a clinically confirmed diagnosis of IIH, and the availability of MRI examinations with sufficient diagnostic quality. The diagnosis of IIH was established according to the revised diagnostic criteria for pseudotumor cerebri syndrome proposed by Friedman et al. [1]. Exclusion criteria included patients older than 18 years, inadequate MRI image quality at the time of diagnosis, intracranial hypertension secondary to medication, dural sinus thrombosis, and the presence of systemic diseases known to be associated with intracranial hypertension. A flowchart illustrating patient selection is provided in Figure 1.

A control group frequency-matched to the IIH group by age and sex was included for comparison of PVS scores. Control subjects were selected from pediatric patients who underwent brain MRI for non-specific clinical indications, including headache, strabismus, and endocrine disorders such as growth hormone deficiency, and who demonstrated no evidence of intracranial pathology, hydrocephalus, or imaging findings suggestive of intracranial hypertension. None of the control subjects had a clinical diagnosis of IIH or documented signs of raised intracranial pressure.

Demographic data, including age and sex, were recorded for all participants. Patients with IIH were categorized as prepubertal or pubertal according to clinical records and chronological age assessment. BMI and age- and sex-specific BMI percentiles were recorded for all patients with IIH. In the control group, BMI and BMI percentiles were available for 34 of 46 participants (73.9%), whereas pubertal status could not be reliably determined.

Lumbar puncture with CSF opening pressure measurement was performed in all 46 patients with IIH, and the measurements were obtained from clinical records. Lumbar puncture opening pressure was measured in the lateral decubitus position, without sedation whenever feasible; sedation was reserved for younger children when clinically necessary. Thirty-one of 46 patients (67.4%) underwent lumbar puncture under sedation. Elevated intracranial pressure was defined according to the revised Friedman criteria as a CSF opening pressure ≥28 cm H_2_O in obese or sedated children, or ≥25 cm H_2_O in non-obese, non-sedated children [1]. The median interval between MRI and lumbar puncture was 2 days (IQR, 1–11.5 days). MRI was performed before cerebrospinal fluid drainage in 36 of 46 patients, whereas lumbar puncture preceded MRI in 10 patients.

### 2.2. MRI Acquisition

MRI examinations in both the IIH and control groups were performed using the same 1.5-T scanner (Philips Ingenia, Philips Medical Systems, Eindhoven, The Netherlands) with the same standard pediatric brain MRI protocol. The protocol included axial and coronal T2-weighted, axial FLAIR, axial T1-weighted, diffusion-weighted, susceptibility-weighted, and sagittal 3D T1-weighted sequences. Axial T2-weighted images were acquired with TR/TE of 6000–6500/100–140 ms, FOV of 160–200 mm, an acquisition matrix of 512 × 512, corresponding to an in-plane resolution of approximately 0.31–0.39 × 0.31–0.39 mm^2^, a slice thickness of 5 mm, and a 1 mm interslice gap. When available, contrast-enhanced MRV, phase-contrast MRV, or postcontrast sagittal 3D T1-weighted sequences were used for evaluation of the dural venous sinuses. Venous imaging was available in 40 of 46 patients, including contrast-enhanced venous imaging in 27 patients and phase-contrast MR venography in 13 patients.

### 2.3. Imaging Analysis

All MRI examinations were evaluated by two experienced pediatric radiologists (with 10 and 8 years of experience in pediatric neuroradiology, respectively). Imaging findings associated with IIH—including empty sella, optic nerve tortuosity, optic nerve sheath distension, posterior globe flattening, and optic disc protrusion—were initially assessed independently by the two radiologists. Disagreements regarding these conventional MRI findings were resolved by consensus; when consensus could not be reached, a third pediatric radiologist adjudicated the finding.

Transverse sinus stenosis was defined as unilateral or bilateral focal luminal narrowing, typically involving the lateral portion of the transverse sinus, relative to the adjacent normal-caliber sinus segment, and was recorded as unilateral or bilateral. Diffuse, uniformly reduced caliber of the transverse sinus was considered developmental hypoplasia and was not classified as stenosis. Source images and multiplanar reconstructions were reviewed in addition to MIP images, with contrast-enhanced images evaluated when available to distinguish true stenosis from flow-related signal loss. Disagreements were resolved by consensus and, when necessary, by a third radiologist.

PVS assessment was performed independently by both observers, who were not blinded to group allocation, on axial T2-weighted images using the Potter grading system [15]. For the primary analyses, PVS scores assigned by the more experienced reader were used, while scores from both readers were used to assess interobserver agreement. PVS were identified based on their characteristic morphology and CSF-like signal characteristics, with FLAIR and T1-weighted images used to distinguish potential mimics. At the level of the centrum semiovale and basal ganglia, at least three consecutive slices were evaluated, and the slice demonstrating the highest PVS burden was selected to minimize slice selection bias. PVS were counted separately for each cerebral hemisphere, and the higher score was recorded for analysis. PVS were graded as follows: grade 0 (none), grade 1 (1–10), grade 2 (11–20), grade 3 (21–40), and grade 4 (>40). For basal ganglia assessment, regions at and below the level of the anterior commissure were excluded from scoring. At the level of the brainstem, PVS were assessed as absent (grade 0) or present (grade 1).

The frequency of radiological findings was compared between prepubertal and pubertal patient groups. PVS scores were compared between the IIH and control groups. Additionally, the relationship between CSF opening pressure and PVS scores was analyzed. The association between TSS and PVS scores was also assessed.

### 2.4. Statistical Analysis

All statistical analyses were performed using IBM SPSS Statistics for Windows, version 23.0 (IBM Corp., Armonk, NY, USA). Continuous variables were expressed as mean ± standard deviation or median (interquartile range), as appropriate according to data distribution. Categorical variables were summarized as frequencies and percentages.

Comparisons between two independent groups were performed using the independent samples *t*-test for normally distributed variables and the Mann–Whitney U test for non-normally distributed variables. Categorical variables were compared using the chi-square test or Fisher’s exact test, as appropriate. Holm correction was applied to multiple comparisons of conventional MRI findings according to TSS status. Effect sizes for nonparametric comparisons were calculated using rank-biserial correlation and reported with 95% confidence intervals.

PVS scores between the IIH and control groups were compared using the Mann–Whitney U test. Holm correction was applied to account for multiple testing across the three regional PVS comparisons. A secondary ordinal logistic regression analysis was performed to assess whether the observed group difference in PVS burden remained after adjustment for BMI, age, and sex. Differences in the frequency of radiological findings between prepubertal and pubertal groups were analyzed using the chi-square test.

Interobserver agreement for PVS grading was assessed in the entire study cohort using quadratically weighted Cohen’s kappa for ordinal centrum semiovale and basal ganglia scores and standard Cohen’s kappa for binary brainstem assessment. Agreement was interpreted as poor (κ < 0.20), fair (0.21–0.40), moderate (0.41–0.60), good (0.61–0.80), or excellent (0.81–1.00).

Correlation analyses between CSF opening pressure and PVS scores were performed using Spearman’s rank correlation coefficient. The association between TSS and PVS scores was evaluated using the Mann–Whitney U test.

Centrum semiovale PVS scores were compared among TSS-positive patients, TSS-negative patients, and controls using the Kruskal–Wallis test, followed by pairwise Mann–Whitney U tests with Holm correction.

Associations of BMI percentile with regional PVS burden and TSS status were also evaluated.

A two-tailed *p*-value of <0.05 was considered statistically significant.

## 3. Results

A total of 67 pediatric patients with raised intracranial pressure and suspected IIH were identified during the study period. After application of the exclusion criteria, 46 patients with clinically confirmed IIH were included in the final analysis (Figure 1). The IIH and control groups were similar in age (11.8 ± 3.3 vs. 11.8 ± 3.3 years) and sex distribution (female: 52.2% vs. 54.3%, respectively). Demographic characteristics and frequencies of radiological findings are summarized in Table 1.

Among the radiological findings associated with IIH, TSS was the most common abnormality, identified in 25 of 40 patients (62.5%) with available venous imaging.

No statistically significant differences were observed between prepubertal and pubertal patients regarding radiological findings or cerebrospinal fluid opening pressure. Twenty-nine patients (63.0%) had a BMI at or above the 85th percentile, compared with 8 of 34 controls (23.5%) with available BMI data. BMI was higher in the IIH group than in controls with available BMI data (*p* = 0.003). BMI percentile was not significantly correlated with PVS scores in the centrum semiovale (ρ = −0.087, *p* = 0.566), basal ganglia (ρ = 0.175, *p* = 0.246), or brainstem (ρ = −0.045, *p* = 0.764; *n* = 46). BMI percentile did not differ significantly between patients with and without TSS (*p* = 0.585).

After Holm correction for multiple comparisons, patients with TSS demonstrated significantly higher frequencies of empty sella (adjusted *p* < 0.001), optic nerve tortuosity (adjusted *p* = 0.038), and optic nerve sheath distension (adjusted *p* = 0.021) compared with patients without TSS.

Overall interobserver agreement for PVS grading was good for the centrum semiovale (weighted κ = 0.663, 95% CI, 0.550–0.776) and basal ganglia (weighted κ = 0.622, 95% CI, 0.467–0.777), and moderate for the brainstem (κ = 0.595, 95% CI, 0.429–0.761).

**Figure 2:**
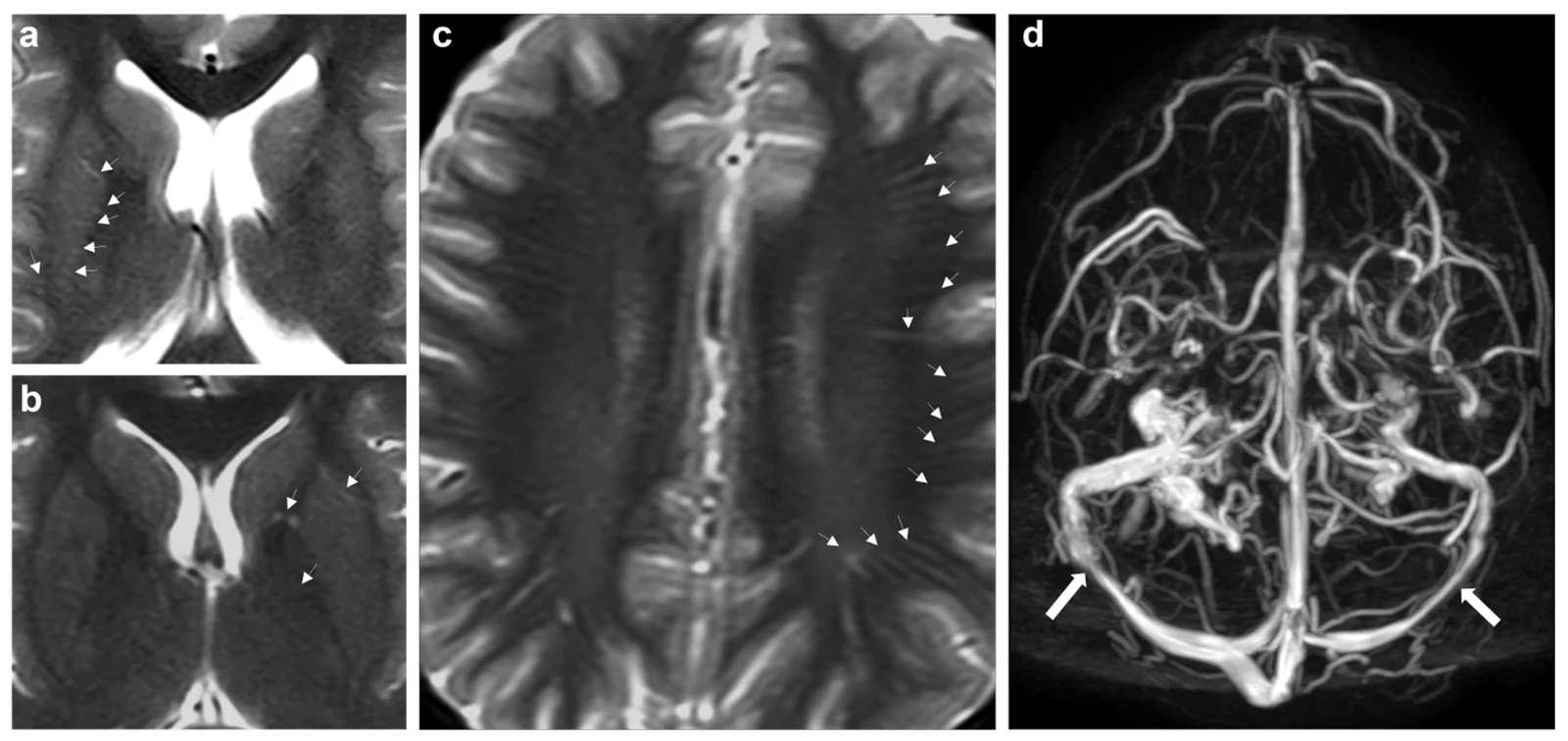
PVS burden and TSS in pediatric IIH. Axial T2-weighted images at the level of the basal ganglia demonstrate grade 2 PVS burden in a pediatric patient with IIH (**a**) compared with grade 1 PVS burden in a control subject (**b**). In panels (**a**–**c**), arrowheads indicate MRI-visible PVS. In another patient with IIH, axial T2-weighted imaging at the centrum semiovale level (**c**) demonstrates a high PVS burden (grade 3), while corresponding MR venography maximum intensity projection (MIP) image (**d**) demonstrates bilateral transverse sinus stenosis (long arrows).

Basal ganglia PVS scores were significantly higher in pediatric patients with IIH than in controls (unadjusted *p* = 0.005; Holm-adjusted *p* = 0.014) with a moderate effect size (rank-biserial correlation = 0.30, 95% CI, 0.10–0.49). This association remained significant after adjustment for BMI, age, and sex (adjusted OR, 3.61; 95% CI, 1.30–10.01; *p* = 0.014). No significant differences were observed in centrum semiovale or brainstem PVS scores (Table 2). Representative MRI findings are shown in Figure 2.

CSF opening pressure did not differ significantly between patients who underwent lumbar puncture with and without sedation (median, 40 vs. 38 cmH_2_O; *p* = 0.870). No significant correlations were observed between CSF opening pressure and PVS scores (centrum semiovale: ρ = 0.117, *p* = 0.440; basal ganglia: ρ = 0.116, *p* = 0.444; brainstem: ρ = 0.035, *p* = 0.819; *n* = 46). Centrum semiovale PVS scores differed according to TSS status, with higher scores in TSS-positive than TSS-negative patients (Holm-adjusted *p* = 0.007). However, TSS-positive patients did not differ significantly from controls, whereas TSS-negative patients had lower scores than controls (Holm-adjusted *p* = 0.007) (Table 3).

## 4. Discussion

In this case–control MRI study, we assessed regional PVS burden in pediatric patients with IIH compared with age- and sex-matched controls and evaluated its association with conventional radiological findings and TSS. The main finding of our study was a significantly higher basal ganglia PVS burden in pediatric patients with IIH compared with controls. Within the IIH cohort, centrum semiovale PVS burden differed according to TSS status; TSS-negative patients had lower PVS scores than both TSS-positive patients and controls, whereas TSS-positive patients did not differ significantly from controls.

Recent interest in the glymphatic system has led to increasing investigation of MRI-visible PVS as a potential imaging marker of alterations in CSF and interstitial fluid exchange [6,11,16]. In adult IIH populations, several studies have reported an increased burden of visible PVS, supporting the hypothesis that elevated intracranial pressure and impaired venous drainage may influence glymphatic clearance pathways [11]. However, findings in pediatric patients remain limited and inconsistent. In contrast to the findings reported in adults, Cohen et al. found no significant differences in PVS burden between pediatric patients with IIH and controls [12]. In our cohort, only basal ganglia PVS scores were significantly higher in the pediatric IIH group compared with controls. The basal ganglia are supplied by numerous perforating lenticulostriate vessels and may therefore be more sensitive to subtle changes in perivascular fluid dynamics [5,17]. Accordingly, the increased PVS burden observed in this region may indicate that glymphatic alterations in pediatric IIH are regionally heterogeneous rather than diffuse.

In our cohort, centrum semiovale PVS burden differed according to TSS status: TSS-negative patients had lower PVS scores than both TSS-positive patients and controls, whereas TSS-positive patients did not differ significantly from controls. Previous studies have proposed that impaired venous outflow may influence glymphatic or interstitial fluid clearance [8], and the centrum semiovale contains prominent medullary venous drainage pathways that may render PVS visibility in this region susceptible to alterations in venous hemodynamics [8,16]. TSS is also recognized as a common and diagnostically relevant MRI finding in IIH [18]. Nevertheless, our findings suggest that the relationship between TSS and MRI-visible PVS may be more complex than initially hypothesized and do not provide direct evidence that venous outflow impairment results in increased PVS burden. Interestingly, conventional MRI findings of IIH, including empty sella, optic nerve sheath distension, and optic nerve tortuosity, were more frequently observed in patients with TSS, indicating a distinct radiological phenotype within the IIH cohort. The relationship between TSS and intracranial pressure remains incompletely understood, and venous sinus narrowing may represent either a contributing factor or a secondary phenomenon in different patient subgroups [6,19,20,21]. Accordingly, the observed association between TSS status and regional PVS burden should be considered exploratory and interpreted without implying a causal relationship.

Notably, no significant correlation was identified between CSF opening pressure and PVS scores in any evaluated region. A similar observation was reported in a previous pediatric study [12]. This finding may indicate that MRI-visible PVS burden is not solely determined by the degree of intracranial pressure elevation. Instead, factors such as chronic pressure changes, impaired venous drainage, or altered tissue compliance may influence PVS visibility more than the degree of intracranial pressure itself. Recent reviews have similarly emphasized that glymphatic alterations in IIH may represent dynamic or adaptive changes rather than a simple direct consequence of elevated intracranial pressure [6]. Therefore, MRI-visible PVS burden may reflect factors beyond the degree of acute intracranial pressure elevation, although the underlying mechanisms remain uncertain.

Our study has several limitations. First, the retrospective single-center design may limit generalizability. BMI was higher in the IIH group than in controls, and BMI data were not available for all control participants; however, the association with basal ganglia PVS burden remained significant after adjustment for BMI, age, and sex. Pubertal status could not be reliably determined in the control group, and detailed metabolic variables were not systematically available, precluding adjustment for these potential confounders. Second, PVS evaluation was performed using a semiquantitative visual grading system rather than automated volumetric analysis or imaging techniques that directly assess glymphatic function. Therefore, the potential relationship between MRI-visible PVS and glymphatic alterations remains inferential. In addition, PVS grading was performed without blinding to group allocation, which may have introduced observer bias. Third, the use of 1.5-T MRI scanners may have limited visualization of small perivascular spaces compared with higher-field-strength imaging systems. In 10 patients, lumbar puncture preceded MRI, and a potential effect of CSF drainage on subsequent MRI findings cannot be excluded. In addition, MR venous imaging was not available in all patients and was not available in the control group, precluding assessment of TSS status in controls and potentially introducing selection bias in TSS analyses. Different imaging techniques were also used for TSS evaluation. The relatively small TSS-negative subgroup (*n* = 15) limits the precision of subgroup comparisons, and these findings should therefore be interpreted with caution. Despite these limitations, our study also has several strengths. We included an age- and sex-matched control group, compared PVS burden between groups, systematically evaluated conventional imaging findings in the IIH cohort, and demonstrated good interobserver agreement for PVS grading. In addition, all MRI examinations were obtained before IIH-specific treatment, minimizing the potential influence of treatment on imaging findings. Our findings contribute to the limited pediatric literature by showing higher basal ganglia PVS burden in pediatric IIH compared with controls.

## 5. Conclusions

In conclusion, pediatric idiopathic intracranial hypertension was associated with increased basal ganglia perivascular space burden, suggesting a regionally heterogeneous pattern of PVS alterations. Centrum semiovale PVS burden differed according to TSS status, with lower scores in TSS-negative patients than in both TSS-positive patients and controls, whereas TSS-positive patients did not differ significantly from controls. These findings may reflect alterations in perivascular fluid dynamics, although their relationship with glymphatic function remains to be clarified. Further studies using advanced imaging techniques and longitudinal follow-up are needed to better understand the mechanisms underlying PVS alterations in pediatric idiopathic intracranial hypertension.

## Figures and Tables

**Table 1:** Demographic and radiological characteristics of pediatric patients with idiopathic intracranial hypertension.

Variable	Value
Number of patients	46
Age, mean ± SD (years)	11.8 ± 3.3
Female sex, *n* (%)	24 (52.2)
Male sex, *n* (%)	22 (47.8)
Prepubertal patients, *n* (%)	12 (26.1)
Pubertal patients, *n* (%)	34 (73.9)
Empty sella/pituitary flattening, *n* (%)	19 (41.3)
Optic nerve tortuosity, *n* (%)	19 (41.3)
Optic nerve sheath distension, *n* (%)	19 (41.3)
Posterior globe flattening, *n* (%)	7 (15.2)
Optic disc protrusion, *n* (%)	4 (8.7)
Transverse sinus stenosis ^a^, *n* (%)	25 (62.5)
CSF opening pressure, median (IQR), cmH_2_O	39.0 (32.3–57.8)

^a^ Evaluated in 40 patients with available venous imaging. CSF, cerebrospinal fluid; IQR, interquartile range; SD, standard deviation.

**Table 2:** Comparison of PVS grade distribution between pediatric IIH patients and controls.

Region/Grade	IIH (*n* = 46) *n* (%)	Control (*n* = 46) *n* (%)	Unadjusted *p* Value
**Centrum semiovale**			0.434
Grade 0	0	0	
Grade 1	6 (13.0)	4 (8.7)	
Grade 2	18 (39.1)	16 (34.8)	
Grade 3	21 (45.7)	26 (56.5)	
Grade 4	1 (2.2)	0	
Median (IQR)	2 (2–3)	3 (2–3)	
**Basal ganglia**			0.005 *
Grade 0	0	0	
Grade 1	15 (32.6)	28 (60.9)	
Grade 2	29 (63.0)	18 (39.1)	
Grade 3	2 (4.4)	0	
Grade 4	0	0	
Median (IQR)	2 (1–2)	1 (1–2)	
**Brainstem**			0.087
Grade 0	13 (28.3)	21 (45.7)	
Grade 1	33 (71.7)	25 (54.3)	
Median (IQR)	1 (0–1)	1 (0–1)	

* *p* values were calculated using the Mann–Whitney U test. After Holm correction for multiple regional comparisons, the basal ganglia difference remained statistically significant (adjusted *p* = 0.014). Statistically significant (*p* < 0.05). PVS, perivascular spaces; IIH, idiopathic intracranial hypertension; IQR, interquartile range.

**Table 3:** Centrum semiovale PVS scores according to TSS status and controls.

Group	*n*	Median (IQR)	Pairwise Comparison	Adjusted *p* Value
**TSS present**	25	3 (2–3)	TSS present vs. TSS absent	0.007
**TSS absent**	15	2 (1–2)	TSS absent vs. controls	0.007
**Controls**	46	3 (2–3)	TSS present vs. controls	0.464

Overall Kruskal–Wallis *p* = 0.003. Pairwise comparisons were performed using Mann–Whitney U tests with Holm correction. PVS, perivascular spaces; TSS, transverse sinus stenosis; IQR, interquartile range.

## Data Availability

The data presented in this study are available from the corresponding author upon reasonable request. The data are not publicly available due to privacy and ethical restrictions.
